# Barriers and facilitators for general practitioners to engage in advance care planning: A systematic review

**DOI:** 10.3109/02813432.2013.854590

**Published:** 2013-12

**Authors:** Aline De Vleminck, Dirk Houttekier, Koen Pardon, Reginald Deschepper, Chantal Van Audenhove, Robert Vander Stichele, Luc Deliens

**Affiliations:** ^1^End-of-Life Care Research Group, Ghent University & Vrije Universiteit Brussel (VUB), Belgium; ^2^LUCAS (Center for Care Research and Consultancy), Catholic University of Louvain, Belgium; ^3^Heymans Institute, Ghent University, Belgium; ^4^Department of Public and Occupational Health, and EMGO+ Institute for Health and Care Research, and VU University Medical Centre, The Netherlands

**Keywords:** Advance care planning, barriers, Belgium, facilitators, general practice, general practitioner, systematic review

## Abstract

**Objective:**

The aim of this systematic review is to identify the perceived factors hindering or facilitating GPs in engaging in advance care planning (ACP) with their patients about care at the end of life.

**Design:**

Studies from 1990 to 2011 were found in four electronic databases (PubMed, CINAHL, EMBASE, PsycINFO); by contacting first authors of included studies and key experts; and searching through relevant journals and reference lists. Studies were screened, graded for quality, and analysed independently by two authors; those reporting the perception by GPs of barriers and facilitators to engagement in ACP were included.

**Results:**

Eight qualitative studies and seven cross-sectional studies were included for data extraction. All barriers and facilitators identified were categorized as GP characteristics, perceived patient factors, or health care system characteristics. Stronger evidence was found for the following barriers: lack of skills to deal with patients’ vague requests, difficulties with defining the right moment, the attitude that it is the patient who should initiate ACP, and fear of depriving patients of hope. Stronger evidence was found for the following facilitators: accumulated skills, the ability to foresee health problems in the future, skills to respond to a patient's initiation of ACP, personal convictions about who to involve in ACP, and a longstanding patient–GP relationship and the home setting.

**Conclusion:**

Initiation of ACP in general practice may be improved by targeting the GPs’ skills, attitudes, and beliefs but changes in health care organization and financing could also contribute.

- GPs can easily engage themselves in advance care planning (ACP) but the incidence of GPs engaging their patients in ACP remains low.
- This review adds to the knowledge in this field by also including studies on ACP discussions, whether or not these discussions result in written advance directives.
- Barriers and facilitators to engage in ACP were related to GP characteristics, perceived patient characteristics, and health care system characteristics.
- Stronger evidence was found for GP skills, GP attitudes, and GP beliefs regarding patients as barriers to engage in ACP.

## Introduction

Consistency between a patient's wishes about end-of-life care and the actual care he/she receives at the end of life is considered an important aspect of both patient-centred care and quality end-of-life care [1–3]. This implies that patients’ preferences regarding end-of-life care must be known before they lose the capacity to make these decisions themselves [4].

Advance care planning (ACP) is defined as a voluntary process of discussion about future treatment and end-of-life care preferences care between an individual, his/her family, and his/her care providers should the individual become incapable of making decisions [5]. This process can result in three main outcomes: an “advance statement”, i.e. a documented statement of the patient's general values and views concerning future care and treatment; and/or an “advance directive” (AD), also known as a living will, i.e. instructions regarding end-of-life care (e.g. the forgoing of specific treatment); and the appointment of a substitute decision-maker in the event of loss of capacity [6]. Internationally, different informal and legal documents related to ACP are used, depending on countries’ specific jurisdiction [7–10].

The advantages of the timely initiating of ACP are well known: it facilitates access to palliative care, stimulates communication between the patient, family, and physicians, and results in greater satisfaction for the patient and the bereaved [11]. In an ageing population, more people will die from serious progressive illnesses, making timely initiation of ACP important [12,13]. General practitioners (GPs) are well placed to encourage and engage in ACP [14,15] and the long-term relationship many patients have with their GP may be a good basis for initiating timely discussion [16,17]. Yet previous research has shown that the incidence of ACP discussions and the completion rate of ADs remain low among the general public and in specific patient populations [18–22]. Only 8% of the general public in England and Wales have completed an ACP document of any kind [23]. Surveys conducted in the USA show that only one-third of adults have an AD expressing their wishes for end-of-life care [24] and even among severely or terminally ill patients, fewer than 50% have an AD in their medical record [25]. In Belgium and the Netherlands GPs discussed ACP with terminally ill patients in a third of all cases and documented the discussion in only 8% (Belgium) and 16% (Netherlands) [26]. Although both patients and physicians support the idea of ACP, these results suggest that certain obstacles still prevail [27–29].

The objective of this systematic literature review is to identify the perceived factors that hinder or facilitate GPs in engaging in ACP with their patients; this has not been studied before, though understanding of these barriers and facilitators is important for the development of interventions and training programmes aimed at facilitating ACP in general practice.

## Material and methods

### Search strategy for the identification of studies

Four electronic databases were searched for studies published in English, French, or Dutch between 1990 and 2011: PubMed, CINAHL, EMBASE, and PsycINFO. A search strategy was developed by ADV and DH for Medline and adapted to each database separately. A combination of controlled vocabulary and free text words was used to search in titles and abstracts: advance care planning, advance directives, advance decision, advance statement, living will, general practice, primary health care, general practitioners, family physicians, primary care, primary practice, and family practice.

The reference list of all identified studies was screened for additional relevant studies. The first author of each included study and known experts in the field of ACP were contacted for more studies. Furthermore, the most recent issues of 10 relevant journals were hand-searched for relevant papers.

### Inclusion and exclusion criteria

An article was included if it reported (1) primary research, (2) on barriers and facilitators (3), on GPs, (4) on patient involvement in ACP. The inclusion criteria are defined as follows:

(1) Primary research: Both quantitative and qualitative studies reporting original data that contain a clearly formulated research question or study aim were included. Editorials, narrative reviews, comments, and expert opinion were excluded.
(2) Barriers and facilitators are conceptualized as predisposing factors, reported by the GP, that hinder or facilitate their engagement in the process of ACP with their patients such as skills, beliefs, and experiences [30].
(3) GPs: Articles reporting on general practitioners, family physicians, or family doctors were included. Where a study reported on various types of health care professionals there must have been separate results for GPs.
(4) ACP is defined as a voluntary process of discussion about future treatment and end-of-life care preferences between an individual, his/her family, and his/her care providers should the individual become incapable of making decisions [5]. Studies reporting only on discussions about future care without involvement of the patient were excluded.

### Inclusion procedure

Duplicates of the retrieved records were removed. Titles and abstracts of all identified reports were screened independently by ADV and DH using a standardized study selection form. The eligibility of selected studies was independently assessed by ADV and DH. Disagreement was resolved by discussion and a third reviewer (KP) was available for arbitration.

### Data extraction

Characteristics of the studies included were extracted to a standardized data-extraction form. ADV and DH independently extracted data under the headings of general information, country, research question, design, participants, barriers and facilitators, and quality assessment scores.

Barriers and facilitators were extracted from the individual studies as mentioned in the article. Factors that were found as barriers and as facilitators in the same article were reported both as a barrier and a facilitator. Factors only reported as barriers or only as facilitators in an article were also categorized only as barriers or facilitators. Discrepancies between reviewers were discussed and if consensus could not be reached, a third reviewer (KP) was consulted.

### Quality assessment and grading evidence

The quality of studies was appraised and evaluated using the Critical Appraisal Skills Programme (CASP) [31]. Since no CASP tool is available for cross-sectional studies, an additional critical appraisal tool developed by Crombie (21-item list) was used [32]. Total quality assessment scores for both qualitative and quantitative studies are presented as scores on a scale from 0 to10.

**Figure 1.**
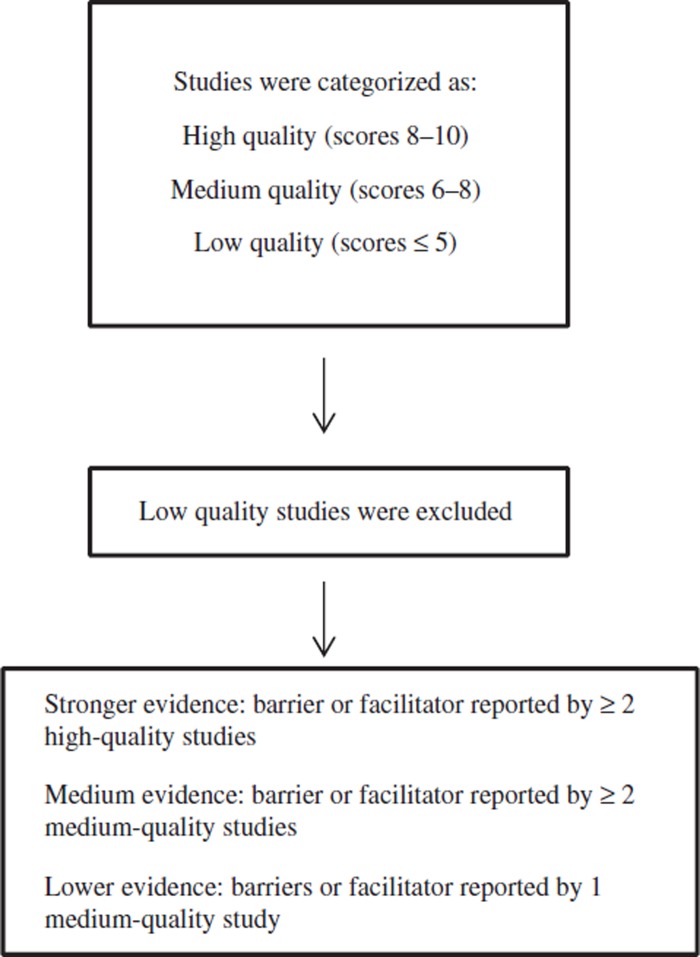
Quality assessment.

In addition, the body of evidence from the multiple studies was graded using the three important elements for grading systems suggested by the Agency for Healthcare Research and Quality: quality, quantity, and consistency [33]. The individual studies were categorized as high-quality studies (scores from 8 to 10), medium-quality studies (scores from 6 to 8), and low-quality studies (scores equal to or lower than 5). Articles with low-quality ratings were excluded from further data extraction. Factors reported in two or more high-quality studies were graded as stronger evidence. Factors reported in one high-quality study or two medium-quality studies were graded as medium evidence and factors reported in one medium-quality study were graded as lower evidence. Consistency of the findings was achieved through the classification of all reported factors as barriers or facilitators, preceding the analysis of the results (Figure 1).

## Results

### Identification of relevant studies (Figure 2)

From the electronic databases searches 442 records were identified. After removal of duplicates and irrelevant reports, the title and abstract of 320 records was screened and 61 full-text articles were retrieved for detailed evaluation. Contact with the first authors of included articles and known experts in the field, a search in reference lists, and hand-searching through relevant journals yielded 42 records. Sixteen articles met all inclusion criteria and were included for data extraction and quality assessment as were nine qualitative studies and seven cross-sectional studies.

**Figure 2.**
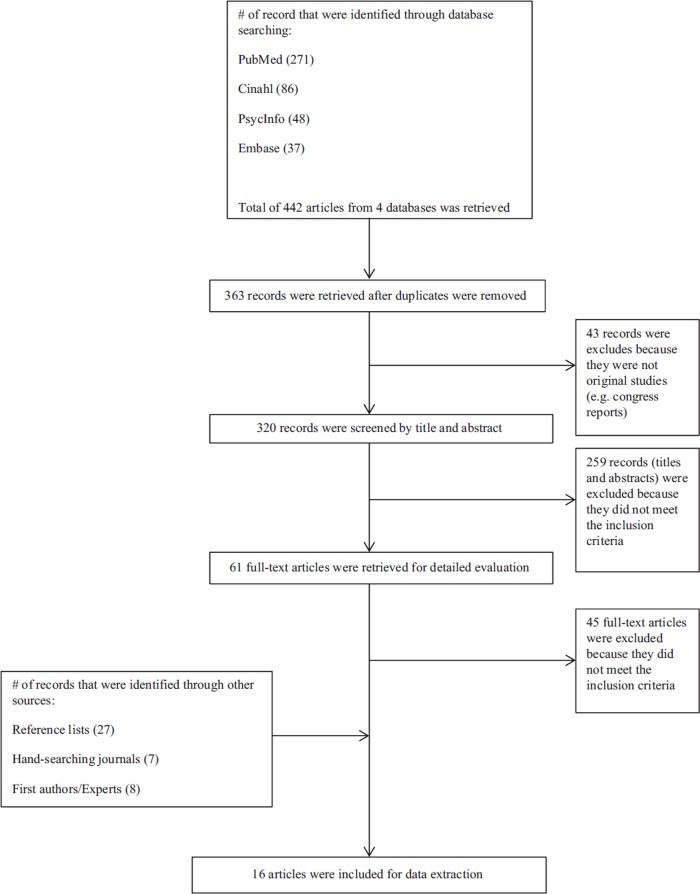
Flow diagram of literature search and selection of articles.

### Characteristics and quality assessment of relevant studies (Table I)

Of the 16 included studies, four were conducted in the USA, four in the UK, two in the Netherlands, two in Australia, and one in Belgium, Canada, Singapore, and Israel. Of the nine qualitative studies, six studies used semi-structured interviews and three studies used both interviews and focus groups. Data in all quantitative studies were collected through questionnaires. Different types of ACP were addressed in the included studies: eight reported on communication about end-of-life care in general, eight others on more specific types of ACP (e.g. ADs).

**Table I.** Characteristics and quality assessment of studies included in the review.^1^

Study	Country	Research question	Design	Participants	Quality assessment^2^
A. Qualitative studies					
1. Minto & Strickland (2011) [40]	UK	To explore the perceptions of GPs and district nurses (DNs) who have experience of ACP for their patients approaching the end of life	Semi-structured interviews	Lead GP (n = 1), GP (n = 1), district nurse (n = 3)	8
2. Boyd et al. (2010) [15]	UK	To explore the views of GPs and community nurses in 4 Scottish practices about ACP for cancer patients; to evaluate their learning objectives; and to see if a tailored educational intervention that could be delivered at practices during continuing education would encourage greater involvement in ACP	Mixed methods (including semi-structured interviews)	General practitioners (n = 20) and community nurses (n = 8)	7,5
3. Munday et al. (2009) [14]	UK	To explore the experiences and perceptions of general practitioners and community nurses in discussing preferences for place of death with terminally ill patients	Semi-structured interviews	General practitioners (n = 17) and nurses (n = 19)	8,5
4. Ostertag & Forman (2008) [64]	USA	To start a community inquiry into concerns at EOL by exploring the opinions and experiences of community members and health care workers	Structured interviews and focus groups	Hospice medical directors (n = 3), hospice nurse managers (n = 4), primary care physicians (n = 14), long-term care facility staff (n = 18), hospice staff (n = 12), community religious leaders (n = 9), members of the volunteer hospice board (n = 9), and family members of patients who died (n = 19)	4,5
5. Bentur (2008) [44]	Israel	To determine what clinicians know about Israel's new “Dying Patient Act” and its recommendations, to examine their attitudes and perceptions about it, and to assess their willingness to increase their involvement in advance care planning	In-depth face-to-face interviews and focus groups	Stakeholders and specialists in the health care system (n = 10), senior family physicians, and geriatricians (n = 40)	6
6. Borgsteede et al. (2007) [38]	Netherlands	To investigate whether patients and their GPs talk about euthanasia and, if so, how they communicate about this	Semi-structured interviews	General practitioners (n = 20) and their patients (n = 30)	8,5
7. Thompson et al. (2003) [42]	UK	To discover the views of health professionals on advance directives	Semi-structured interviews and focus groups	Hospital doctors (n = 4), GPs (n = 4), nurses (n = 4); hospital nurses, hospice staff, GPs, consultant geriatricians, geriatricians in training grades, and an interdisciplinary group (n = 34)	7,5
8. Brown (2002) [41]	Australia	To explore the issues for GPs when introducing advance directives to their patients as a basis for further research into this process	Interviews with GPs before and after the introduction of advance directives to patients during a normal consultation	General practitioners (n = 5)	7
9. Pfeifer et al. (1994) [39]	USA	To identify primary care patients’ and physicians’ beliefs, attitudes, preferences, and expectations regarding discussions of EOL medical care, and to identify factors limiting the quality and frequency of these discussions	Structured, qualitative interviews with open-ended questions	Primary care physicians (n = 43) and ambulatory outpatients (n = 47)	8
B. Quantitative studies					
1. Meeussen et al. (2011) [26]	Belgium	To investigate the prevalence and characteristics of ACP in two European countries and identify the associated factors	Questionnaire, nationwide mortality follow-back study	Non-sudden deaths (n = 1072) (Belgium (n = 755); Netherlands (n = 317)	9
2. James et al. (1998) [43]	USA	To assess the impact of traditionally unmeasured patient-centred factors on primary care physicians’ decisions to adhere to an evidence-based clinical practice guideline for heart failure	Questionnaires	Family physicians (n = 459)	8,5
3. Steinberg et al. (1997) [35]	Australia	To examine health practitioner and community concerns, priorities, and preferred options regarding patient self-determination in terminal care	Postal questionnaire	General practitioners (n = 229)	6,5
4. Tee et al. (1997) [36]	Singapore	To find out the attitudes and to assess the extent of knowledge regarding the AD among general practitioners in Singapore	Cross-sectional, descriptive survey	General practitioners (n = 199)	7
5. Pijnenborg et al. (1994) [34]	Netherlands	To gain insight into decisions made in general practice about the end of life	Analysis of death certificates and questionnaires	Cases in which decisions about the end of life have been made (n = 5197)	8
6. Hughes & Singer (1992) [45]	Canada	To examine the attitudes towards, the experience with and the knowledge of advance directives of family physicians in Ontario	Questionnaire	Family physicians (n = 643)	8,5
7. Doukas et al. (1991) [37]	USA	To determine the degree to which family physicians in the United States discuss and use the living will with their patients	Questionnaire	Family physicians (n = 494)	8,5

Notes: ^1^Research question and study designs are presented as formulated in the articles. ^2^Qualitative studies were appraised by using the critical appraisal tool for qualitative research by CASP (10-item list). Quantitative studies were appraised using the critical appraisal tool for surveys developed by Crombie (21-items list). Both scales were converted to a 10-point scale.

Quality scores ranged from 4.5 to 8.5 for the qualitative studies and from 4.5 to 9 for the quantitative studies, both on a scale of 10. On the basis of these scores, four qualitative studies were considered as high quality, four as medium and one as low (excluded for further data extraction). Of the seven quantitative studies, we appraised five as high-quality studies and two as medium-quality studies.

### Barriers and facilitators for GPs (Table II)

All reported barriers and facilitators were categorized as GP characteristics, perceived patient characteristics, or health care system characteristics.

**Table II.** Barriers and facilitators to GPs engaging in ACP, as reported by the GP.

	Barriers	Facilitators
GP characteristics	Socio-demographic characteristics of GPs / Knowledge of GPs → Lack of GP knowledge about ACP (e.g. about the legal status or time of execution of advance directives) [B3; B4; B7]** Skills of GPs → Dealing with vague requests from patient [A3; A6]*** → Dealing with patients’ changing preferences [A9]** → Dealing with uncertainty of prognosis for cancer patients [A2]* → Difficulties with defining the right moment [A6; B7]*** → Experiencing difficulties with advising patients in expressing their wishes [A8]* → Dealing with emotional impact by the GP [A1]** → Dealing with feeling uncomfortable by the GP [B7]**	Socio-demographic characteristics of GPs →Younger age of the GP [B5]** Knowledge of GPs → Good knowledge about ACP (e.g. regarding the use of advance directives or living wills) [B4; B7]** Skills of GPs → Accumulated skills [A3; B6]*** → Defining the right timing for patients [A3]** → Anticipating health problems in the future [A6; B2]*** → Dealing with patients initiating discussions [A2; A3; A8; A9; B7]*** → Dealing with explicit patient preferences [A3]**
	Experience of GPs /	Experience of GPs → Years of experience as GP [A1]** → Positive experiences with end-of-life conversations in the past [A2]* → GP personally signed living will [B7]**
	GP attitudes → Thinking it is the GP's job to cure people [A9]** → Considering other healthcare professionals better positioned to initiate ACP [A3]** → Patients should initiate discussion [A6; B4; B7]*** → The document of living wills itself is too legalistic and simplified for complicated medical scenarios [A9]** → Doubts about the pragmatic availability of living will documents [A9]**	GP attitudes → Considering ACP as part of the job [A2; A8]** → Perceived usefulness of advance directives [A7; B3; B6]** → Physicians should initiate the discussion [B7]**
Perceived patient characteristics	Patient-related obstacles to initiate ACP discussions → Patient's denial of terminal illness [A3]** → Patients have fear of upsetting their families [B3]* → Patients are reluctant to think about future health care problems [B3]* → Patient lack knowledge of ACP – Lack of patient knowledge about the processes involved in making advance directives [B3]* – Patients not understanding or misinterpreting the GP [B7]** – Complexity and length of the advance directive form is too hard for patients to understand the instructions and complete it [A5]* – Patients’ misunderstanding about the sorts of health problems one will have in the future and the implications of treatment refusal [A7]*	Patient-related facilitators to initiate ACP discussions → Patient's acceptance of terminal illness [A3]** → Patient knowledge about ACP [B6]**
	Anticipated adverse outcomes as a result of ACP discussions → Fear of depriving patients of hope [A2; A9; B5]*** → Fear of harming GP–patient relationship [A9]**	Anticipated adverse outcomes as a result of ACP discussions /
	Personal convictions about which patients not to involve in ACP discussions → Initiating communication with religious patients is difficult [A6]* → Medical condition of patient as barrier for ACP discussions: – Incapacity of patient because of diminished consciousness or dementia [B5]**	Personal convictions about which patients to involve in ACP discussions → According to medical condition of patient: - Chronically ill patients or specific life-threatening diagnoses (e.g. cancer, end-stage heart disease) [B6]** - Short-term prognosis or terminally ill patients [B3; B4; B6]** – Cancer patients are more involved in ACP discussions as opposed to non-cancer patients [A6; B1]*** – Patients who are competent in decision-making [B1; B5]*** – Healthy patients (e.g. all adults, all patients over 65 years of age) [B4; B6]** → When do ACP discussions take place: – Admission or discharge from hospital [A2; B6]** – End-of-life decisions that are estimated to shorten patients’ life by more than one week [B5]** – Patients receiving palliative care [B1]**
Health care system characteristics	Related to the GP practice → Time limitations [A8]* → Limited resources available to honour patients’ or families’ expectations [A1] **	Related to the GP practice → Long-term GP–patient relationship – Shared common history [A3; A6]*** – Multiple contacts in the last week of life [B1]** → Time – Possibility to devote time [A3]** – Reimbursement of time spent on discussion [B6]** → Care setting – Home setting [A6; B1]*** – GP office [B4]*
	Related to other health care providers → Lack of collaboration with secondary care – Patients reaching the end of their lives leaving the care of their family physician [A5]* – Patients getting different messages from the hospital and the GP [A2]* Related to legislation /	Related to other health care providers → Collaboration with other healthcare professionals – Respect of advance directives by other healthcare professionals [B3]* – Consultation with other health care professionals about ELDs [B5]** Hospital policy supporting or requiring the use of advance directives [B6]** – Related to legislation → Legislation regarding advance directives – Legalization supporting the use of advance directives [B3; B6]** – Legalization protecting physicians when following advance directives [B6]**

Notes: *** = Stronger evidence; ** = Medium evidence; * = Lower evidence.

### GP characteristics

*Socio-demographic characteristics of GPs.* There was medium evidence that the GP being younger was significantly and positively associated with the proportion of patients with whom they discussed end-of-life decisions [34].

*Knowledge.* Medium evidence was found for the reported lack of GP knowledge about ACP as a barrier to involving patients in ACP [35–37].

*Skills.* There is stronger evidence that GPs perceive their own lack of skill in dealing with patients’ vague requests, and their difficulties in defining the right moment for initiating discussion, as barriers to engaging in ACP [14,37,38]. Medium evidence was found that they perceive their lack of skill in dealing with a patient's changing preferences and with the emotional impact or discomfort of having ACP discussions as barriers [37,39,40]. Lower evidence supported the perceived lack of skill in advising patients on expressing their wishes, and the prognostic uncertainty for chronically ill patients, as barriers [15,41]. Addressing patient initiation, accumulated skills, and foreseeing health problems in the near future were factors reported as facilitators for which stronger evidence was found [14,15,39,41–43].

*Experience.* Medium evidence was found for the length of their experience as a GP and having a living will themselves as perceived facilitators [37,40]. Lower evidence supported positive experiences with end-of-life conversations in the past as a facilitator [15].

*Attitudes.* The attitude that GPs should initiate ACP was perceived as a facilitator for which stronger evidence was found [37]. There is medium evidence that a conviction that it is their job to cure people whereas other healthcare professionals should initiate ACP prevents GPs engaging in ACP [14,39]. Doubts regarding the content and practical availability of living wills are hindering factors as well [39].

### Perceived patient characteristics

*Perceived patient-related obstacles* can hinder GPs in initiating ACP. The GP holding the following beliefs is perceived as a barrier and supported by lower evidence: patients lack knowledge of ACP, patients have a fear of upsetting their families, and patients are reluctant to think about future health care problems [35,37,42,44]. Medium evidence supports that a patient's denial of his/her terminal illness makes talking about preferences for end-of-life care very difficult [14].

*Anticipated adverse outcomes.* Fear of depriving a patient of hope or damaging the GP–patient relationship were cited as factors that keep GPs from engaging in the process of ACP, for which respectively stronger and medium evidence was found [15,34,39].

*Personal convictions about who and who not to involve in ACP and when*. When asked who should be approached about ACP, GPs designated terminally ill patients and healthy patients in medium-quality studies [15,35,36,45]. GPs reporting that competent patients and cancer patients are more involved in ACP is supported by stronger evidence [26,34,38]. Medium evidence was found that three events trigger discussion between GPs and patients: admission or discharge of patients from hospital, when end-of-life decisions are estimated by the GP to shorten patients’ life by more than one week, and when patients receive treatment aimed at palliation in the last week of life [15,26,34,45].

### Health care system characteristics

*Related to the GP practice*. Stronger evidence supported a longstanding patient–GP relationship as a perceived facilitator for ACP [14,38]. GPs also considered it advantageous if talking about ACP could take place in the home setting [26,38]. There is medium evidence for the time available, and the chances of reimbursement, being facilitators [14,45]. The limited resources available in primary care were perceived as a barrier [40].

*Related to other healthcare providers*. There is lower evidence that lack of collaboration with secondary care is perceived as an impediment to the process of ACP [15,44]. Consultation with other healthcare professionals and hospital policy supporting or requiring the use of ADs was considered as a facilitator, supported by medium evidence [34,45].

*Related to legislation*. GPs reported that legislation supporting the use of ADs as well as protecting GPs who follow them would encourage them to offer ADs to patients, which is supported by medium evidence [35,45].

## Discussion

We found numerous perceived barriers and facilitators influencing GP engagement in ACP with patients. All reported factors were related to three groups: GP characteristics, perceived patient characteristics, and health care system characteristics. Stronger evidence was found for lack of skills to deal with vague requests, difficulties with defining the right moment, the attitude that patients should initiate ACP, and fear of depriving them of hope as perceived barriers. The perceived facilitators for which stronger evidence was found were accumulated skills, the ability to foresee health problems in the future, skills in addressing patient initiation of ACP, cancer patients, patients capable of decision-making, a longstanding patient–GP relationship, and a home setting.

To our knowledge, this is the first study to provide a systematic overview of the perceived barriers to and facilitators for GPs engaging in ACP. All required methodological steps to complete a systematic review were implemented and performed separately by two reviewers. This review adds to the knowledge in this field by also including studies on ACP discussions, whether or not these discussions result in written advance directives [46].This review also has limitations. Given the variation in how ACP is implemented and documented and the variation in GP practice, our findings may not be generalizable to all countries and health care systems. Second, only barriers and facilitators reported by GPs were considered although understanding the barriers and facilitators for patients is equally important and deserves research. Third, we retrieved only qualitative research and observational studies, though in our opinion such research designs provide the best way of addressing the research question. As the studies used different methods, it was not appropriate to combine data across the studies for meta-analysis [47,48]. However, the methodological quality was assessed and, in addition, the body of evidence was graded. This approach allows for provision of a conclusion that incorporates both the results and quality of the studies [49].

Stronger evidence was found for the GP attitude that patients should initiate discussions being a barrier and for having the skill to address a patient's initiation of discussion as a facilitator. Remarkably, many studies show that patients believe it is the physician's responsibility to initiate ACP, which suggests that there is a gap in expectation between patients and GPs. This difference has been pointed out in previous studies and may explain why ACP consultations are often initiated tardily when end-of-life decisions need to made [50–53].

Most of the perceived barriers for GPs were classified as a lack of skills; it is recognized that physicians are less likely to initiate ACP when they believe they lack the skills required [54]. The perceived lack of skills to deal with a patient's changing preferences and to address vague requests and difficulties with defining the right moment to initiate ACP were also found in other health care settings [28,55]. Many of the same barriers could also be found in the literature on communication at the end of life in general and may cover the same ground, since ACP is often initiated at the end of life [56–58].

According to GPs, cancer patients are more involved in the process of ACP than non-cancer patients. As they often have a more predictable disease course, defining the right moment to initiate ACP might be easier. Research has shown that one of the reasons ACP was not initiated with patients with chronic obstructive pulmonary disease (COPD) was because of physicians’ lack of understanding that COPD is a life-threatening disease [21]. Not only physicians but also chronically ill non-cancer patients often have a poor understanding of their illness [59,60]. It is possible that non-cancer patients initiate ACP less often because of a reduced awareness of their diagnosis and prognosis. Most patients and professionals agree that talking about ACP should take place around the time of diagnosis of a life-threatening illness, but fear of depriving patients of hope is a barrier preventing GPs from initiating ACP for which stronger evidence was found [61,62].

The facilitators identified were often related to health care system characteristics. Previous research showed that conversations about ADs averaged 5.6 minutes and physicians spoke for two-thirds of this time, making patient's values and preferences rarely explored [63]. Financial compensation for the time spent on ACP could possibly encourage GPs to make ACP a current practice but it could also acknowledge the importance of this aspect of care.

Understanding the barriers and facilitators is important for the development of interventions aimed at facilitating ACP in general practice. Initiation of ACP in general practice may be improved by targeting GP-related barriers and facilitators, but changes in health care organization and finances could also contribute. Training programmes are necessary to change skills, attitudes, and beliefs preventing GPs from initiating ACP and to provide good role models for the difficult task of initiating communication about end of life in a helpful and empathetic way.
